# Laparoscopic colectomies associated with decreased retrieval of 12 or more lymph nodes compared to open in elective colon cancer surgery

**DOI:** 10.3332/ecancer.2019.968

**Published:** 2019-10-14

**Authors:** Yana Puckett, Diana Mitchell, Theophilus Pham

**Affiliations:** Department of General Surgery, Texas Tech University Health Sciences Center, Lubboc, TX 79430, USA

**Keywords:** Colon cancer, lymph nodes, laparoscopic colectomy

## Abstract

**Background:**

Colorectal cancer is the third most commonly diagnosed cancer worldwide. Lymph node (LN) retrieval is a key factor for pathologic staging and prognosis of colorectal cancer. Increase in number of LNs removal suggests improvement in tumour clearance and decrease in metastatic spread. Studies have suggested that excising 12 or more LNs during colectomy in patients with colon cancer is associated with improved survival. To date, there have been no studies to determine whether minimally invasive surgery affects the ability to retrieve 12+ LNs in elective colon cancer surgery. Therefore, we elected to determine whether a difference exists on the ability to retrieve 12+ nodes in elective colon cancer colectomies performed open versus laparoscopic.

**Methods:**

The National Surgical Quality Improvement Program (NSQIP) Procedure Specific Colectomy database was analysed for the year 2014–2015. Inclusion criteria were colon cancer (ICD-9 Code 153.9), age greater than 18 years. Exclusion criteria were missing data. Data abstracted included patient demographics, type of operation performed and number of LNs retrieved. The patients were categorised based on their elective colon cancer colectomies such as laparoscopic or open. Binary logistic regression was used to identify confounding variables in the retrieval of 12+ LNs.

**Results:**

After accounting for missing cases, a total of 18,792 patients with a diagnosis of colon cancer were analysed. Twelve or more LNs were retrieved in 88% (16,538) of patients, Among them, 2,516 patients underwent laparoscopic colectomy and 5,284 patients underwent open colectomy. The difference was not statistically significant for the average number of LNs retrieved among both the groups. Open operative approach compared to the laparoscopic approach was associated with 15% greater odds of retrieval of >12 LNs (OR 1.148; 95% CI (1.035–1.272); *p* = 0.008).

**Conclusion:**

The majority of colectomies such as open or laparoscopic are able to retrieve 12 or more LNs. However, there are greater odds of retrieving more than 12 LNs with the open approach compared to the laparoscopic approach. By allowing for more LN retrieval, open colectomies suggest improvement in tumour clearance and decrease metastatic spread. Additional research is needed to further investigate the specific factors influencing the ability to retrieve an adequate number of LNs, such as viewing angles provided with an open approach versus laparoscopic approach.

## Introduction and background

Colorectal cancer is the third most commonly diagnosed cancer worldwide [1]. Surgery is considered a basic treatment plan for most patients with colorectal cancer and the primary element of any curative treatment plan. It is no surprise, that key factor in the care of colon cancer patients is to perform the colon cancer operation. Lymph node (LN) retrieval is a key factor for pathologic staging and prognosis of colorectal cancer. Cancer can be staged using the TNM system, where the T plus a letter or number (0–4) is used to indicate whether the primary tumour has grown into the bowel lining. >T2 indicated that the tumour has grown through the layers of the colon. The N in the TNM system stands for LNs. N1 indicates that the tumour is found in less than three regional LNs, and N2 indicates that the tumour is found in more than four regional LNs. The M in the TNM system describes metastasis.

Increase in number of LNs removal suggests improvement in tumour clearance and decrease in metastatic spread [2]. Therefore, excision of sufficient number of LNs during resection of colorectal cancer is crucial. Variable confounding factors may influence the level of LN retrieval. The patient demographics, type of tumour and treatment factors have been key factors to determine the number of LNs to be retrieved in colon cancer. Surgical extent has also been to be known influence on number of LN retrieval. However, many factors can influence the excision of LNs in colorectal cancer treatment such as training and experience of treating doctors; patients demographics (age and gender specifically); type of surgical to be performed; tumour invasion; location of the tumour; size of the tumour; stage of the tumour; resected bowel segment length and preoperative therapies [6].

Several studies have shown an inverse correlation between the number of positive LNs and survival. There is independent and positive association between an increase in the number of LNs excision and improved survival rate for both node-positive and node-negative patients, while a higher lymph node ratio (LNR) is correlated with increased mortality, overall and cancer-specific [2, 3].

An excision of 12 or more LNs is considered as the current standard of treatment in colorectal carcinomas [2, 4]. The National Comprehensive Cancer Network (NCCN) and the College of American Pathologists, and the American Joint Committee on Cancer (AJCC) suggest a minimum of 12 LNs to establish the colorectal staging [19]. The probability of harvesting more LNs has been predicted by a more contemporary year of operation, increased tumour stage, higher tumour grade, younger patients and non-Caucasian ethnicity [2]. Harvesting fewer LNs has been associated with the year of operation, early tumour stage, elder patients and the operation approach [4]. Although in recent years, the proportion of patients having at least 12 LNs examined has increased significantly, there is still a need to increase the percentage of adequate LN retrieval (only 80% in 2011) [2, 4].

Most studies achieved greater than 12 LNs using various procedures, such as laparoscopic complete mesocolic excision (CME), open CME, CME with complete vascular ligation, laparoscopic radical LN dissection including non-regional LNs, robotic surgery, intra- and extra-corporeal laparoscopic surgery, single- and multi-port laparoscopic surgery, 2D versus 3D laparoscopic surgery and even a multidisciplinary approach [4–15].

Laparoscopic surgery has effectively replacing open colonic surgery in recent times with favourable short-term outcomes, such as minimal pain, reduced blood loss and improved survival rate [20]. Laparoscopic colon surgery is different from other types of intra-abdominal laparoscopic surgeries and it is considered as an advanced surgical procedure. These operations take place in multiple quadrants. Initially, there was a concern regarding the cancer recurrence after laparoscopic colectomy. However, in several studies, patients with colon cancer were randomly assigned to undergo either open or laparoscopic colectomies, where both laparoscopic surgery and open surgery were correlated with similar disease-free and survival status [21].

Many studies have assessed different surgical procedures for colon cancer resection and indicated the mean number of harvested LNs. Some studies when comparing laparoscopic-assisted and open mesocolic excision found that there is no difference in number of harvested LNs and oncologic clearance between the two groups [6, 15]. Other studies found that the mean number of LNs retrieval is greater in open CME [9]. When comparing laparoscopic colectomy and open colectomy, there has been no significant difference in number of LNs resected [9, 16], and single-port versus multi-port laparoscopic techniques have also shown no significant difference in terms of number of harvested LNs [13, 17].

Up until now, there have been no studies to determine whether minimally invasive surgery affects the ability to retrieve 12+ LNs in elective colon cancer surgery. Therefore, we elected to determine whether a difference exists on the ability to retrieve 12+ nodes in elective colon cancer colectomies performed open versus laparoscopic.

## Methods

The National Surgical Quality Improvement Program (NSQIP) Procedure Specific Colectomy database was analysed for the year 2014–2015. Patients diagnosed with colon cancer (ICD-9 Code 153.9) with age greater than 18 years were included in the study. Patients with age less than 18 years, patients with other diagnoses than colon cancer or patients with missing data were excluded from the study. Robotic colectomies were excluded from the analysis. The patients were categorised based on their elective colon cancer colectomies such as laparoscopic colectomy or open colectomy.

Data were tested for homogeneity utilising histogram plots. Data appeared to be homogeneous and removed outliers. Data abstracted included patient demographics, type of operation performed, number of LNs retrieved and TNM staging. Independent *t*-test and Chi-square test were used to compare factors between the groups, where independent *t*-test was used for continuous variables and Chi-Square test was used for categorical variables. Binary logistic regression was used to identify confounding variables in the retrieval of 12+ LNs. IBM SPSS software, V.22.0 (SPSS, Armonk, NY, USA) was used to perform statistical analysis. Statistical significance was set at a *p*-value of 0.05.

## Results

After accounting for missing data cases, a total of 18,792 patients with a diagnosis of colon cancer were analysed. Greater than 12 LNs were retrieved in 88% (16,538) of patients and the remaining 12% (2,254) patients had less than 12 LNs retrieval. The overall mean number of LNs for open colectomy patients was 20 (SD 10) versus 19 (SD 9) for laparoscopic colectomies (*p* = 0.546). Overall, laparoscopic colectomies were able to retrieve >12 LNs in 85% of the cases while open colectomies were able to retrieve >12 LNs in 90.3% of the cases (Table 1).

Among greater than 12 LNs retrieved patients, 2,516 underwent laparoscopic colectomy and 5,284 underwent open colectomy (Table 2).

The difference was not statistically significant for average number of LNs retrieved among the patients who underwent laparoscopic or open colectomy. Open operative approach compared to laparoscopic was associated with 15% greater odds of retrieval of >12 LNs [OR 1.148; 95% CI (1.035–1.272); *p* = 0.008]; >T2 status [OR 1.273; 95% CI (1.148–1.413); *p* < 0.0001]; and metastatic colon cancer [OR = 1.194; 95% CI (1.013–1.408); *p* = 0.035]. Patients that underwent chemotherapy within 90 days of colectomy had a 16% less chance of retrieval of >12 LNs [OR = 0.834; 95% CI (0.723–0.963); *p* = 0.013].

## Discussion

Adequate LNs retrieval is a crucial factor for pathologic staging and prognosis of colorectal cancer, it also suggests improvement in tumour clearance and decrease in metastatic spread. A retrospective study found that the proportion of patients who had at least 12 LNs retrieved increased by a statistically significant extent between 1997 and 2013. They evaluated that this improvement might be due to multiple factors which made surgeons aware of the significance of LN examination for colon cancer suffering patients [6]. Upon analysis of the NSQIP database, patients who underwent an open operative approach had slightly greater odds of retrieval of greater than 12 LNs when compared to patients who had a laparoscopic operation.

This study utilised a national database to gather data on the number of LNs retrieved, while other studies utilised retrospective analysis of charts [15], prospectively collected databases [8], retrospective analysis of regional discharge records [16], and PubMed and MEDLINE database searches [6]. It is difficult to compare between studies because of discrepancies in sampling methods, likely account for differences in inclusion criteria and patient demographic characteristics which likely contribute to inconsistencies between the studies. As the NSQIP is a national database, the cases included in this study capture a broad range of patients, and smaller studies using retrospective chart analysis may have patients with less variety in their demographics leading to inconsistencies between the studies. Finally, the large sample size drawn from the NSQIP, compared to the smaller sample sizes of other studies, likely contributes to dissimilarities between studies and the statistical power of results.

This study is limited by the retrospective nature of the data collection. A previous study found that the proportion of patients with <12 LNs excised were increased with age. Also, the tumour stage demonstrated a statistically significant association with the number of LNs retrieved. The proportion of patients with <12 excised LNs were higher in the early-stage of colorectal cancer. However, the process of LN examination during the curative resection of colon cancer has improved [4]. By drawing data from the NSQIP, we were limited to the variables available for analysis, and, therefore, confounding variables such as age, cancer stage, operative approach, location of cancer in the colon, etc., that may affect the usefulness in MOABP in LNs retrieval were not analysed. Without access to this information, we were unable to case match and account for patient characteristics that may be affecting the utility of MOABP on LNs retrieval. Finally, there is the possibility of errors in data input, but with such a large sample size, the effect of such errors should be minimal.

For conventional colorectal cancer surgery, LN retrieval plays an important role in the evaluation of patient survival and determining the requirement for adjuvant therapy. Higher numbers of LN retrieval is associated with increased survival rate for both node-positive and node-negative patients [2, 4]. The laparoscopic approach has developed quickly with increased number of studies reporting its safety and efficacy versus an open approach. With its rapid development, there is a concern that this new technology may limit the removal of cancerous LNs; however, several randomised-controlled trials (RCTs) have reported no significant difference in the number of LNs harvested between a laparoscopic and an open approach. A study reported that laparoscopic surgeries are feasible and safe in elderly patients with increased level of comorbidity. There was no significant worsening of intra- and postoperative outcomes after laparoscopic colorectal procedures. Some authors compared laparoscopic colorectal surgery in older patients with open surgery and substantially suggested that the minimally invasive procedures had a greater benefit on them. Few studies indicated that laparoscopy is a surgically safe and acceptable treatment approach for well-selected patients with colon cancer to ensure faster short-term recovery of the patients [9, 16].

Until now, there have been no studies to determine whether minimally invasive surgery affects the ability to retrieve 12+ LNs in elective colon cancer surgery. Hence, the aim of this study was to determine whether a difference exists on the ability to retrieve 12+ nodes in elective colon cancer colectomies performed open versus laparoscopic. We determined that there are greater odds of retrieving more than 12 LNs with open approach compared to laparoscopic. Additional research is needed to further investigate the specific factors influencing the ability to retrieve an adequate number of LNs, such as viewing angles provided with an open approach versus laparoscopic approach.

## Conclusion

In conclusion, the retrieval of greater than 12 LNs in colorectal cancer colectomies is associated with better staging and better prognosis for the patient. Our results based on a national database in the United States show that both open and laparoscopic colectomy approaches in colorectal cancer were associated with the retrieval of greater than 12 LNs, on average. However, open colectomy approach was found to be an independent predictor of retrieval of greater than 12 LNs. Additional research is needed to further investigate the specific factors influencing the ability to retrieve an adequate number of LNs, such as viewing angles provided with an open approach versus a laparoscopic approach.

## Conflicts of interest

The authors declare that they have no conflicts of interest.

## Funding declaration

No external funding was used for this study.

## Figures and Tables

**Table 1.** Comparison of retrieval of lymph nodes by operative method for colorectal cancer colectomies.

Variables	Mean (SD)	<12 nodes retrieved (*n* = 2,254)	>12 nodes retrieved (*n* = 16,538)	*p*-value
Laparoscopic colectomy (*n* = 2,960)	19.0 (9.0)	15.00% (444)	85.00% (2,516)	0.084
Open colectomy (*n* = 5,852)	20.0 (11.0)	9.71% (568)	90.29% (5,284)	0.009

**Table 2.** Multivariate logistic regression of predictors of retrieval of greater than 12 lymph nodes in colorectal cancer colectomies.

Variables	<12 nodes retrieved (*n* = 2,254)	>12 nodes retrieved (*n* = 16,538)	Odds ratio	95% confidence interval	*p*-value
Open colectomy (*n* = 5,852)	9.71% (568)	90.29% (5,284)	1.148	1.035–1.272	0.008
Neoadjuvant chemotherapy within 90 days of operation	19.74% (445)	10.71% (1,771)	0.834	0.723–0.963	0.013
>T2	42.99% (969)	62.95% (10,410)	1.273	1.148–1.413	<0.0001
N0	48.80% (1110)	55.88% (9,241)	0.976	0.883–1.080	0.642
N1	20.98% (473)	25.43% (4,205)	1.040	0.853–1.295	0.602
N2	7.19% (162)	12.8% (2,118)	1.098	0.943–1.278	0.230
Mets	7.85% (177)	6.00% (993)	1.194	1.013–1.408	0.035
